# Analysis of Site-Specific Methylation of Tumor-Related Genes in Head and Neck Cancer: Potential Utility as Biomarkers for Prognosis

**DOI:** 10.3390/cancers10010027

**Published:** 2018-01-22

**Authors:** Kiyoshi Misawa, Daiki Mochizuki, Atsushi Imai, Masato Mima, Yuki Misawa, Hiroyuki Mineta

**Affiliations:** Department of Otolaryngology/Head and Neck Surgery, Hamamatsu University School of Medicine, Shizuoka 431-3192, Japan; daiki_m525@yahoo.co.jp (D.M.); imaimimi@yahoo.co.jp (A.I.); tendoon@gmail.com (M.M.); mswyuki@abox3.so-net.ne.jp (Y.M.); mineta@hama-med.ac.jp (H.M.)

**Keywords:** epigenetic regulation, cancer metastasis, tumor-related genes, head and neck cancer, site-specific analysis

## Abstract

Clarifying the epigenetic regulation of tumor-related genes (TRGs) can provide insights into the mechanisms of tumorigenesis and the risk for disease recurrence in HPV-negative head and neck cancers, originating in the hypopharynx, larynx, and oral cavity. We analyzed the methylation status of the promoters of 30 TRGs in 178 HPV-negative head and neck cancer patients using a quantitative methylation-specific PCR. Promoter methylation was correlated with various clinical characteristics and patient survival. The mean number of methylated TRGs was 14.2 (range, 2–25). In the multivariate Cox proportional hazards analysis, the methylation of *COL1A2* and *VEGFR1* was associated with poor survival for hypopharyngeal cancer, with hazard ratios: 3.19; *p* = 0.009 and 3.07; *p* = 0.014, respectively. The methylation of *p16* and *COL1A2* were independent prognostic factors for poor survival in laryngeal cancer (hazard ratio: 4.55; *p* = 0.013 and 3.12; *p* = 0.035, respectively). In patients with oral cancer, the methylation of *TAC1* and *SSTR1* best correlated with poor survival (hazard ratio: 4.29; *p* = 0.005 and 5.38; *p* = 0.029, respectively). Our findings suggest that methylation status of TRGs could serve as important site-specific biomarkers for prediction of clinical outcomes in patients with HPV-negative head and neck cancer.

## 1. Introduction

Head and neck squamous cell carcinoma (HNSCC) is a disease with a high incidence and an anatomically heterogeneous group of solid tumors affecting mainly the oral cavity, pharynx (naso-, oro-, and hypopharynx) and larynx [1]. Major risk factors for HNSCC include sex, tobacco smoking, alcohol consumption, and an oncovirus infection [2]. In recent decades, the overall incidence of HNSCC has been declining in the developed world due to a reduction in the consumption of tobacco. However, there is a concomitant increase in the incidence of oropharyngeal cancer as a result of human papillomavirus (HPV) infection [3,4]. Additionally, HPV-related tumors more frequently arise in the oropharynx, whereas HPV-negative tumors are more common in the hypopharynx, larynx, or oral cavity [5].

Interestingly, HPV-related HNSCC is a distinct clinical entity, with a significantly improved treatment response and survival rates in comparison to HPV-negative HNSCC [6]. In general, the 5-year overall survival for HPV-negative HNSCC is around 50%, while that for HPV-related HNSCC patients is around 80% [7,8]. The biological mechanisms underlying the different outcomes in HPV-negative versus HPV-related HNSCC remain poorly understood. It is, therefore, critically important to find biomarkers for HPV-negative HNSCC in order to facilitate patient stratification and improve treatment outcomes.

Aberrant promoter methylation is considered a major mechanism underlying the inactivation of tumor-related genes (TRGs). Notably, the methylation profile of gene promoters is different for each type of tumor, allowing the identification of patterns of tumor-specific hypermethylation [9]. Global DNA methylation profiling has revealed that HPV-related HNSCC is a unique molecular entity that exhibits hypermethylation compared to HPV-negative tumors [10,11,12]. It is important to keep in mind that the molecular spectrum of HNSCC reflects strong influences of what appear to be significantly different tumor microenvironments [12]. Therefore, the development of an integrated analysis method, applicable to various tumor types, is necessary to understand the correlation between a primary tumor site and tumor-specific characteristics.

The aim of this study was to determine the methylation status of TRGs to evaluate their clinical significance as prognostic biomarkers for survival and risk for recurrence in HPV-negative HNSCC. In an attempt to determine if these DNA methylation events are specific to anatomical sites, we have evaluated and compared the methylation changes originating in different anatomical sites (hypopharynx, larynx, and oral cavity). This site-specific analysis may serve as a valuable resource to determine biomarkers for prediction of clinical outcomes in patients with HPV-negative HNSCC.

## 2. Results

### 2.1. Characteristics of Patients

The clinicopathological data of the 178 HNSCC patients included in the study are summarized in Table 1. The study population comprised of 153 (86%) men and 25 (14%) women with an age range of 32–92 years (mean ± SD.: 65.8 ± 11.2 years). While 61 (34%) patients had hypopharyngeal cancer, 49 (28%) had laryngeal cancer, and 68 (38%) had oral cavity cancer in the head and neck region. The rates of smoking and alcohol drinking were both 75%. Most patients (75%) had an advanced stage disease (III/IV) at diagnosis, and positive recurrence events were noted in 67 cases (38%) (Table 1).

### 2.2. Analysis of Methylation Status of Tumor-Related Genes

**Figure 1:**
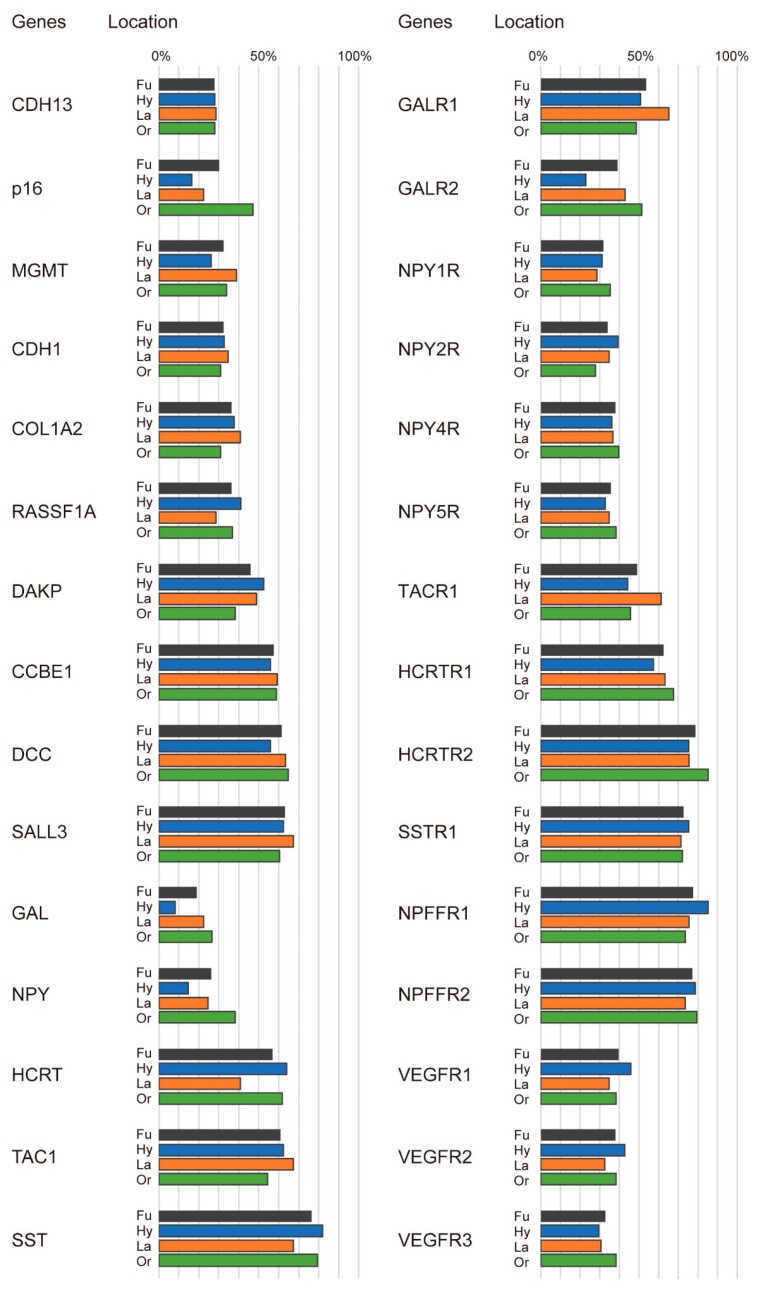
Summary of hypermethylation of tumor-related gene (TRG) promoters in HNSCC samples. Shown here are the methylation frequencies (%) of 30 TRGs in the cohort. Grey bars: Fu: full panel; blue bars: Hy: hypopharyngeal cancer; orange bars: La: laryngeal cancer; green bars: Or: oral cavity cancer.

The frequencies of the promoter hypermethylation of the 30 genes are shown in Figure 1. The mean number of methylated genes in the full panel was 14.3 (range, 2–25). The overall frequencies of promoter methylation indicated that five genes (*SST*, *SSTR1*, *HCRTR2*, *NPFFR1*, and *NPFFR2*) were frequently methylated (greater than 70%), while three genes (*GAL*, *NPY*, and *CDH13*) were less frequently methylated (less than 30%). We also performed detailed stratified analyses to determine the distributions of methylation status based on the original cancer site. This analysis revealed that methylation frequencies of *SST*, *SSTR1*, *HCRTR2*, *NPFFR1*, and *NPFFR2* genes were greater than 70% in the hypopharynx, larynx, and oral cavity. Notably, the less frequently methylated genes (less than 30%) were as follows: *CDH13*, *p16*, *MGMT*, *GAL*, *NPY*, *GALR2*, and *VEGFR3* for hypopharyngeal cancers; *CDH13*, *p16*, *RASSF1A*, *GAL*, *NPY*, and *NPY1R* for laryngeal cancers; and *CDH13* and *GAL* for oral cavity cancers (Figure 1). The methylation status of the 30 tumor-related gene promoters was determined in an additional 516 HNSCC samples and 50 normal samples. The average β values for *p16*, *COAL1A2*, *DAPK*, *CCBE1*, *DCC*, *SALL3*, *NPY*, *TAC1*, *SST*, *GALR1*, *GALR2*, *NPY1R*, *NPY2R*, *NPY4R*, *NPY5R*, *TACR1*, *HCRTR1*, *HCRTR2*, *SSTR1*, *NPDDR1*, *NPFFR2*, *VEGFR1*, *VEGFR2*, and *VEGFR3* methylation were significantly higher in the HNSCC samples than in the normal samples (*p* < 0.05). Methylation of the *CDH13*, *MGMT*, *CDH1*, *RASSF1A*, *GAL*, and *HCRT* promoters was not associated between HNSCC and normal control group. (Figure S1).

### 2.3. Correlation Between Gene Methylation and the Original Tumor Site

Table 2 shows the correlation between the methylation status of TRGs and the original tumor site. Methylation of the *p16* gene had a significantly higher frequency in oral cavity cancers when compared to hypopharyngeal and laryngeal cancers (*p* < 0.001 and *p* = 0.006, respectively). Methylation of the *GAL* gene had a significantly lower frequency in hypopharyngeal cancers when compared to laryngeal cancers and oral cavity cancers (*p* = 0.035 and *p* = 0.007, respectively). Patients with oral cavity cancers showed a significantly higher *NPY* methylation in comparison to patients with hypopharyngeal cancers (*p* = 0.003). We also found significantly lower methylation of the *HCRT* gene in laryngeal cancers compared to hypopharyngeal cancers and oral cavity cancers (*p* = 0.016 and *p* = 0.025, respectively). The *GALR2* gene methylation was significantly lower in hypopharyngeal cancers compared to laryngeal cancers and oral cavity cancers (*p* = 0.026 and *p* < 0.001, respectively) (Table 2).

### 2.4. Correlation Between TRG Methylation and Clinicopathological Assessment

**Figure 2:**
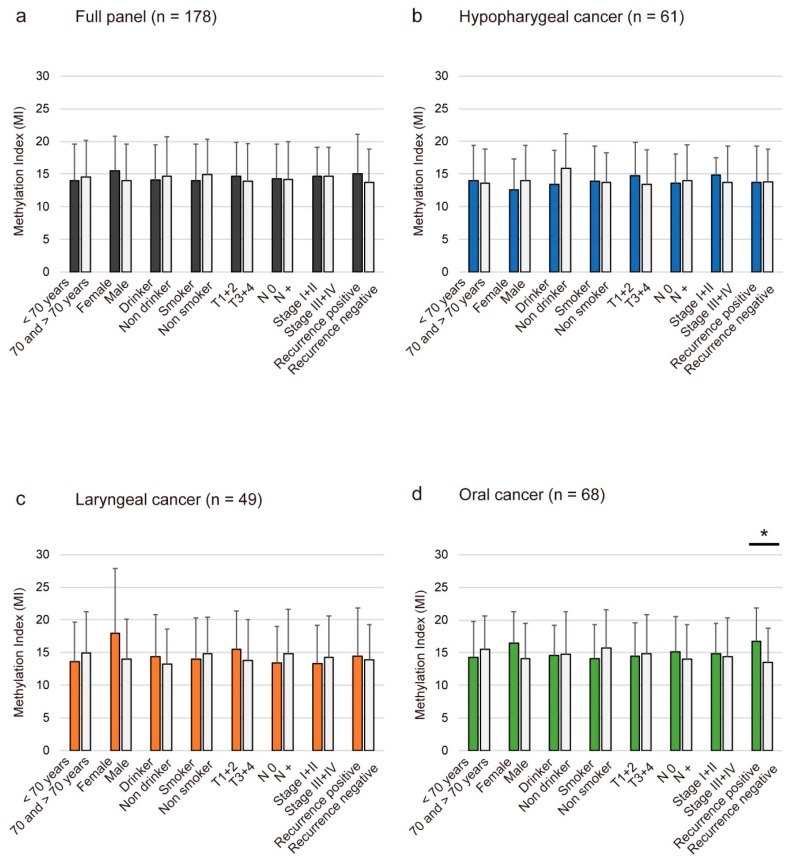
Association between methylation indices (MI) and selected clinical parameters. The mean MI for the various groups was compared using Student's *t*-tests. Shown are the associations between MI and selected epidemiologic and clinical characteristics in (**A**) full panel; (**B**) hypopharyngeal cancer; (**C**) laryngeal cancer; and (**D**) oral cavity cancer. Statistical comparisons between the groups are represented as a mean with standard deviation. A probability of <0.05 (* *p* < 0.05) was considered to represent a statistically significant difference.

Methylation index (MI) was defined as the ratio between the number of methylated genes and the total number of tested genes in each sample. The mean differences in MI according to the age of onset, sex, alcohol consumption, smoking habit, tumor size, lymph node status, clinical stage, and recurrence are illustrated in Figure 2. Continuous marker methylation analyses showed no association between the MI for the 30 TRGs and any clinical parameters in the full panel of 178 patients (Figure 2A) or just in patients with hypopharyngeal (Figure 2B) and laryngeal cancers (Figure 2C). Notably, we found that the MI was significantly higher in the recurrence-positive cases (16.7 ± 5.1) compared to the recurrence-negative cases (13.5 ± 5.2; *p* = 0.017) of oral cancers (Figure 2D).

### 2.5. Associations Between TRGs Methylation and Survival

**Figure 3:**
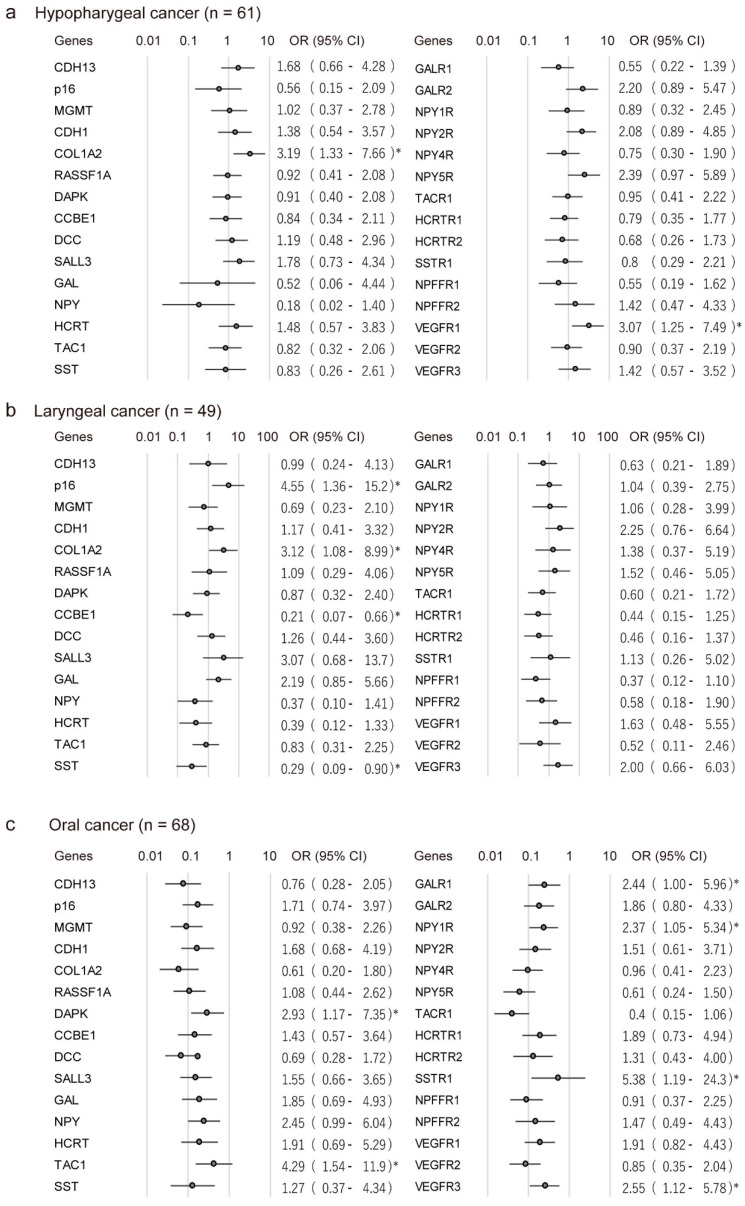
Odds ratios (ORs) for recurrence of cancers. Shown are the ORs of recurrence associated with methylation of different tumor-related genes (TRGs) in (**a**) hypopharyngeal cancer; (**b**) laryngeal cancer; and (**c**) oral cancer. ORs were calculated based on the Cox proportional hazards model, adjusted for age (≥70 years vs. <70 years), sex, alcohol exposure, smoking status, and tumor stage (I and II vs. III and IV). CI: confidence interval. * *p* < 0.05.

The association between methylation and risk of recurrence was estimated via a multivariate analysis using a Cox proportional hazards model adjusted for age (≥70 years vs. <70 years), sex, alcohol consumption, smoking status, and clinical stage. In patients with hypopharyngeal cancers, methylation of *COL1A2* and *VEGFR1* promoters correlated positively with recurrence (odds ratio (OR) = 3.19, 95% CI: 1.33–7.66, *p* = 0.009 and OR = 3.07, 95% CI: 1.25–7.49, *p* = 0.014, respectively) (Figure 3A). In patients with laryngeal cancers, methylation of *p16* and *COL1A2* promoters were associated with poor survival (OR = 4.55, 95% CI: 1.36–15.2, *p* = 0.013 and OR = 3.12, 95% CI: 1.08–8.99, *p* = 0.035, respectively). The opposing influences of promoter methylation of *CCBE1* and *SST* showed association with the OR for recurrence (OR = 0.21, 95% CI: 0.07–0.66, *p* = 0.007 and OR = 0.29, 95% CI: 0.09–0.90, *p* = 0.033, respectively) (Figure 3B). In patients with oral cavity cancers, hypermethylation of *DAPK*, *TAC1*, *GALR1*, *NPY1R*, *SSTR1*, and *VEGFR3* was associated with significantly reduced survival, with hazard ratios of 2.93 (95% CI: 1.17–7.35), 4.29 (95% CI: 1.54–11.9), 2.44 (95% CI: 1.00–5.96), 2.37 (95% CI: 1.05–5.34), 5.38 (95% CI: 1.19–24.3), and 2.55 (95% CI: 1.12–5.78), respectively (Figure 3C).

## 3. Discussion

This study reports a real-time PCR analysis of DNA methylation profiles obtained from the genomic DNA of 178 HNSCC tissues derived from cancers originating in three anatomical sites. Overall, we found that aberrant promoter methylation patterns of specific TRGs are indicators of an increased risk of recurrence. Therefore, the development of an integrated analysis method, applicable to various tumor types, is necessary to determine the correlation between the primary tumor site and the tumor-specific characteristics.

Interestingly, we found a strong association between the methylation levels of *COL1A2* and disease-free survival (DFS) in hypopharyngeal and laryngeal cancers, but not in oral cavity cancers. *COL1A2* is a fibrillar collagen found in most connective tissues and is the main component of the organic part of bones [13]. Hypermethylation of *COL1A2* has been described in breast carcinomas [14], melanomas [15], and medulloblastomas [16,17]. The *COL1A2* methylation status may, therefore, serve as an important site-specific biomarker for the prediction of clinical outcomes in patients with hypopharyngeal and laryngeal cancers.

In laryngeal cancers, the OR for recurrence is higher when the *p16* promoter is methylated versus unmethylated. On the other hand, there is no association between *p16* methylation and prognosis in hypopharyngeal and oral cavity cancers. The *p16* promoter hypermethylation is a widespread epigenetic alteration, which is known to play a significant role in activating *p16* in many tumor types [9,18,19]. Although our results were consistent with these reports, there was a discrepancy in the anatomical subtype. The association of HPV-positive oropharyngeal cancer with methylation levels of the *p16* promoter regions is still controversial. Nakagawa et al. have reported a very low frequency of *p16* promoter region hypermethylation by pyrosequencing. Non-small cell lung cancer patients with hypermethylation of the *p16* promoter are at a moderate risk of recurrence and death in all populations considered [20,21]. Our data, plus that of others, therefore suggest that *p16* methylation affects tumor behavior and clinical outcomes through interaction with tobacco exposure.

In patients with oral cavity cancers methylation of some genes, including *DAPK*, *NPY*, *TAC1*, *GALR1*, *NPY1R*, *NPY2R*, *SSTR1*, and *VEGFR3* correlated with poor survival. We have carefully reviewed the literature on the association between methylation of TRGs and survival in patients with HNSCC (Table 3) [22,23,24,25,26,27,28,29,30,31,32,33,34,35,36]. Oral cavity cancer is a multifactorial disease in which chronic alcohol and tobacco use constitute two major risk factors, while chronic inflammation, viral infections, betel quid/areca-nut chewing, and genetic predisposition are supplementary factors that contribute towards its pathogenesis [37]. Chronic inflammation of the oral mucosa is another risk factor that can potentially modify the methylation status of various genes in oral cavity cancer tumors [38]. The occurrence of multiple cytosine-phosphate-guanine (CpG) methylation sites in a panel of TRGs in oral cavity cancer was highly associated with the stage of cancer progression [32]. Recently, it is reported that saliva-derived DNA is a surrogate noninvasive biomarker panel to discriminate healthy controls from patients with HNSCC [39,40,41]. Therefore, epigenetic alterations may contribute to the etiology of oral carcinogenesis by transcriptional silencing.

Generally, HPV-negative HNSCCs were more broadly distributed among different anatomical sites, compared to the HPV-related tumors, and commonly occurred in the context of heavy alcohol or tobacco use [42]. Despite continuous efforts to identify molecular markers for early detection, and to develop effective treatments, the survival and prognosis of HNSCC patients remain poor [43]. Locoregional recurrence and metastasis are the limiting factors for successful treatments [43]. Profiling DNA methylation is a widely applied tool to identify subtypes of cancers and to predict therapy outcomes [44]. Several studies have been carried out to explore the association between changes in DNA methylation and survival of patients with hypopharynx, larynx, and oral cavity cancers [22,23,24,25,26,27,28,29,30,31,32,33,34,35,36]. As we proceed towards personalized and precision medicine, it is important to remember that, though the genetic material is identical in every cell, epigenetics introduces high variability within different tissues and cell types and is affected by environmental factors [45,46]. Our findings, therefore, suggest that such methylation markers could be used in clinical practice to identify patients who may benefit from adjuvant therapy after an initial surgical treatment. Furthermore, HPV-negative HNSCCs originating in different anatomical sites showed some site-specific DNA methylation events.

## 4. Materials and Methods

### 4.1. Tumor Samples

Primary HNSCC samples (*n* = 178) were obtained from patients during surgery at the Department of Otolaryngology, Hamamatsu University School of Medicine (Hamamatsu, Shizuoka, Japan). All patients provided written informed consent, and the study protocol was approved by the Institutional Review Board of the Hamamatsu University School of Medicine. Clinical information, including age, sex, tumor site, smoking habit, alcohol consumption, tumor size, lymph node status, stage grouping, and recurrence events were all obtained from the patients’ clinical records.

### 4.2. Bisulfite Treatment and Quantitative Methylation-Specific PCR (Q-MSP) Analysis

Genomic DNA was extracted from tumor and normal mucosal tissues using the QIAamp DNA MiniKit (Qiagen, Courtaboeuf, France). DNA was subjected to bisulfite treatment, as described previously [47]. The bisulfite-modified DNA was used as a template for fluorescence-based real-time PCR [48]. The amplifications were performed using a TaKaRa Thermal CyclerDice™ Real Time System TP800 (TaKaRa, Tokyo, Japan). The Q-MSP primers for methylated DNA were Q-MSP-*ACTB*-F (5′-TGGTGATGGAGGAGGTTTAGAAGT-3′) and Q-MSP-*ACTB*-R (5′-AACCAATAAAACCTACTCCTCCCTTAA-3′). A standard curve was generated using serial dilutions of universally methylated DNAs (EpiScope™ Methylated HCT116 gDNA; TaKaRa, Tokyo, Japan). We determined the specificity of these primers using serial dilutions of universally unmethylated DNAs (EpiScope® Unmethylated HCT116 DKO gDNA; TaKaRa, Tokyo, Japan). The normalized methylation value (NMV) was defined as follows: NMV = (TRGs-S/TRGs-FM)/(ACTB-S/ACTB-FM), where TRGs-S and TRGs-FM represent TRG methylation levels in the sample and universally methylated DNAs, respectively. *ACTB*-S and *ACTB*-FM correspond to b-actin in the sample and universally methylated DNAs, respectively. To analyze the methylation status of *CDH13* [49], *p16* [50], *MGMT* [51], *CDH1* [50], *COL1A2* [52], *RASSF1A* [50], *DAPK* [51], *CCBE1* [53], *DCC* [54], *SALL3* [48], *GAL* [55], *NPY* [56], *HCRT* [56], *TAC1* [57], *SST* [58], *GALR1* [47], *GALR* [59], *NPY1R* [56], *NPY2R* [56], *NPY4R* [56], *NPY5R* [56], *TACR1* [57], *HCRTR1* [56], *HCRTR2* [56], *SSTR1* [58], *NPFFR1* [56], *NPFFR2* [56], *VEGFR1* [60], *VEGFR2* [60], and *VEGFR3* [60] primers, conditions and cutoff values were used as previously described. A list of the primer sequences from the Q-MSP analysis is shown in Table S1. The HPV status was evaluated using the HPV Typing Set (Takara, Tokyo, Japan), a PCR primer set specifically designed to identify HPV genotypes -16, -18, -31, -33, -35, -52 and -58 in genomic DNA. The PCR HPV Typing Set method was performed according to the manufacturer’s protocol.

### 4.3. Data Analysis and Statistics

The Q-MSP results and patient characteristics were compared using Student’s *t*-tests. The overall methylation value for individual samples was determined by calculating the methylation index (MI), which was then used to determine the overall methylation rate in the individual samples [59]. The MI for each sample was defined as the ratio of the number of methylated genes to the number of total genes tested (i.e., 30). 

For the frequency analysis in the contingency tables, the associations between variables and methylation status were analyzed statistically using the chi-square test. DFS was calculated from the date of the initial treatment to the date of diagnosis of locoregional recurrence or distant metastasis. The prognostic value of the methylation status was assessed by performing multivariate Cox proportional hazards analysis adjusted for age (≥70 years versus <70 years), sex, alcohol intake, smoking status, and tumor stage (I and II versus III and IV). Differences with P < 0.05 were considered significant. All statistical analyses were performed using StatMate IV (ATMS Co. Ltd., Tokyo, Japan).

### 4.4. Systematic Literature Review

A systematic search in the PubMed database using the following terms: methylation AND survival AND hypopharyngeal cancer OR laryngeal cancer OR oral cavity cancers were performed in order to identify studies reporting genes in which the detection of hypermethylation on their promoter region showed a statistically significant association with their use as a biomarker for prognosis (Table 3).

### 4.5. Collection of Publicly Available Data from TCGA

Aberrant DNA methylation data contained in TCGA (available in May 2017) were collected from the MethHC (http://methhc.mbc.nctu.edu.tw/php/index.php) and using the Infinium Human Methylation 450 platform (Illumina, Inc., San Diego, CA, USA) and are expressed as β-values [61]. 

## 5. Conclusions

In conclusion, high throughput epigenetic screening studies suggest differences in the epigenetic profiles of HPV-related and HPV-negative HNSCC, with the former characterized by hypermethylated genes. Better molecular classification of the head and neck tumors is required to provide prognostic as well as mechanistic information to improve patient care. Future planned studies will include a more diverse patient population and a more comprehensive view of the patient backgrounds and environmental factors.

## Figures and Tables

**Table 1:** Clinicopathological data of head and neck squamous cell carcinoma (HNSCC) patients under study.

Patient and Tumor Characteristics	Full Panel (*n* = 178)	Hypopharynx (*n* = 61)	Larynx (*n* = 49)	Oral Cavity (*n* = 68)
*Age*				
Mean ± S.D.	65.8 ± 11.2	66.7 ± 10.2	68.9 ± 8.3	62.9 ± 13.1
*Gender*				
Male	153 (86%)	54 (89%)	47 (96%)	52 (76%)
Female	25 (14%)	7 (11%)	2 (4%)	16 (24%)
*Alcohol exposure*				
Ever	135 (76%)	51 (84%)	40 (81%)	44 (65%)
Never	43 (24%)	10 (16%)	9 (18%)	24 (35%)
*Smoking status*				
Smoker (>1 pack/20 years)	124 (70%)	46 (75%)	35 (71%)	43 (63%)
Smoker (<1 pack/20 years)	9 (5%)	3 (5%)	2 (4%)	4 (6%)
Non-smoker	45 (25%)	12 (20%)	12 (25%)	21 (31%)
*Tumor size*				
T1	16 (9%)	1 (2%)	4 (8%)	11 (16%)
T2	62 (35%)	21 (34%)	7 (14%)	34 (50%)
T3	40 (22%)	19 (31%)	16 (33%)	5 (7%)
T4	60 (34%)	20 (33%)	22 (45%)	18 (27%)
*Lympho-node status*				
N0	81 (46%)	18 (30%)	24 (49%)	39 (57%)
N+	97 (54%)	43 (70%)	25 (51%)	29 (43%)
*Stage*				
I	14 (8%)	0 (0%)	4 (8%)	10 (15%)
II	32 (18%)	9 (14%)	3 (6%)	20 (29%)
III	37 (21%)	16 (26%)	12 (24%)	9 (13%)
IV	95 (53%)	36 (59%)	30 (61%)	29 (43%)
*Recurrence events*				
Positive	67 (38%)	25 (41%)	20 (41%)	22 (32%)
Negative	111 (62%)	36 (59%)	29 (59%)	46 (68%)

**Table 2:** Correlation between tumor sites and methylation status.

Genes	Methylation Status	Hypopharynx (N = 61)	Larynx (N = 49)	Oral Cavity (N = 68)	*p*-Values ^χ^		
					Hy vs. La	La vs. Or	Hy vs. Or
*CDH13*	Methylated	17	14	19			
	Unmethylated	44	35	49	0.935	0.94	0.993
*p16*	Methylated	10	11	32			
	Unmethylated	51	38	36	0.422	0.006 *	0.0002 *
*MGMT*	Methylated	16	19	23			
	Unmethylated	45	30	45	0.16	0.582	0.348
*CDH1*	Methylated	20	17	21			
	Unmethylated	41	32	47	0.833	0.664	0.817
*COL1A2*	Methylated	23	20	21			
	Unmethylated	38	29	47	0.74	0.267	0.414
*RASSF1A*	Methylated	25	14	25			
	Unmethylated	36	35	43	0.176	0.354	0.623
*DAPK*	Methylated	32	24	26			
	Unmethylated	29	25	42	0.717	0.246	0.105
*CCBE1*	Methylated	34	29	40			
	Unmethylated	27	20	28	0.717	0.969	0.723
*DCC*	Methylated	34	31	44			
	Unmethylated	27	18	24	0.425	0.873	0.298
*SALL3*	Methylated	38	33	41			
	Unmethylated	23	16	27	0.582	0.435	0.813
*GAL*	Methylated	5	11	18			
	Unmethylated	56	38	50	0.035 *	0.619	0.007 *
*NPY*	Methylated	9	12	26			
	Unmethylated	52	37	42	0.197	0.117	0.003 *
*HCRT*	Methylated	39	20	42			
	Unmethylated	22	29	26	0.016 *	0.025 *	0.799
*TAC1*	Methylated	38	33	37			
	Unmethylated	23	16	31	0.582	0.159	0.365
*SST*	Methylated	50	33	54			
	Unmethylated	11	16	14	0.077	0.14	0.714
*GALR1*	Methylated	31	32	33			
	Unmethylated	30	17	35	0.127	0.072	0.795
*GALR2*	Methylated	14	21	35			
	Unmethylated	47	28	33	0.026 *	0.357	*p* < 0.001 *
*NPY1R*	Methylated	19	14	24			
	Unmethylated	42	35	44	0.769	0.444	0.618
*NPY2R*	Methylated	24	17	19			
	Unmethylated	37	32	49	0.616	0.435	0.17
*NPY4R*	Methylated	22	18	27			
	Unmethylated	39	31	41	0.942	0.744	0.671
*NPY5R*	Methylated	20	17	26			
	Unmethylated	41	32	42	0.833	0.695	0.519
*TACR1*	Methylated	27	30	31			
	Unmethylated	34	19	37	0.077	0.095	0.88
*HCRTR1*	Methylated	35	31	46			
	Unmethylated	26	18	22	0.531	0.622	0.228
*HCRTR2*	Methylated	46	37	58			
	Unmethylated	15	12	10	0.99	0.181	0.156
*SSTR1*	Methylated	46	35	49			
	Unmethylated	15	14	19	0.638	0.94	0.666
*NPFFR1*	Methylated	52	37	50			
	Unmethylated	9	12	18	0.197	0.809	0.102
*NPFFR2*	Methylated	48	36	54			
	Unmethylated	13	13	14	0.522	0.452	0.92
*VEGFR1*	Methylated	28	17	26			
	Unmethylated	33	32	42	0.235	0.695	0.378
*VEGFR2*	Methylated	26	16	26			
	Unmethylated	35	33	42	0.285	0.535	0.612
*VEGFR3*	Methylated	18	15	26			
	Unmethylated	43	34	42	0.9	0.394	0.297

Hy vs. La: Hypopharynx vs. Larynx; La vs. Or: Larynx vs. Oral cavity; Hy vs. Or: Hypopharynx vs. Oral cavity; ^χ^ Chi square test used to calculate *p*-value. * *p* < 0.05 considered statistically significant, the same as below.

**Table 3:** Published studies of TRG hypermethylation and survival of hypopharyngeal cancer, laryngeal cancer, and oral cavity cancer patients.

Study (Reference.)	Year	Country	Cases	Primary Site	Genes Studied	Significant Association between Methylation and Survival *
Wei DM [22]	2015	China	53	hypopharyx	*DAPK*	Worse survival (*p* = 0.045)
Stephen JK [23]	2010	USA	79	larynx	*HIC1*, *ESR1*	*HIC1* worse survival (*p* < 0.01)
Shen Z [24]	2016	China	104	larynx	*miR-34a*	Worse survival (*p* = 0.023)
Shen Z [25]	2017	China	91	larynx	*Claudin-11*	Worse survival (*p* = 0.007)
Shen Z [26]	2017	China	93	larynx	*SHISA3*	Worse survival (HR = 2.71; 95% CI: 1.024–7.177; *p* = 0.047)
Ogi K [27]	2002	Japan	96	oral cavity	*MINT31*, *MINT1*, *MINT2*, *MINT27*, *p16*, *p15*, *p14*, *DCC*, *DAPK*	*MINT31* worse survival (HR = 3.79; 95% CI: 1.58–9.10; *p* < 0.001)
Long NK [28]	2008	Japan	40	oral cavity	*RECK*	Worse survival (*p* = 0.023)
Taioli E [29]	2009	USA	88	oral cavity	*MGMT*, *CDKN2A*, *RASSF1*	*MGMT* worse survival (HR = 3.49, 95% CI: 1.62–7.52; *p* = 0.001)
Supić G [30]	2009	Serbia	77	oral cavity	*CDH1*, *DAPK*, *MGMT*, *WRN*, *APC*	*CDH1* worse survival (*p* = 0.024)
Huang KH [31]	2009	Taiwan	166	oral cavity	*RASSF1A*, *HIN-1*, *RASSF2A*, *PTEN*	*RASSF1A* worse survival (n = 166; HR = 2.09, 95% CI: 1.25–3.50), *HIN-1* worse survival (n = 116; HR = 2.66; 95% CI: 1.30–5.45)
Supic G [32]	2011	Serbia	47	oral cavity	*DAPK*, *p16*, *RASSF1A*, *APC*, *CDH1*, *RUNX3*, *WIF1*, *MGMT*, *hMLH*	*DAPK* worse survival (HR = 4.11, 95% CI: 1.46–1.56; *p* = 0.007)
Dong Y [33]	2012	China	30	oral cavity	*p16*	*p16* worse survival (*p* = 0.021)
Lin HY [34]	2013	Taiwan	44	oral cavity	*DAPK*, *RASSF1A*, *IRF8*, *SFRP1*	*DAPK* worse survival (HR = 2.83, 95% CI: 1.05–7.63; *p* = 0.042)
Yang CM [35]	2016	Taiwan	86	oral cavity	*SOX21-AS1*	*SOX21-AS1* worse survival (*p* = 0.002)
Ribeiro IP [36]	2016	Portugal	93	oral cavity	*GATA5*, *WT1*, *MSH6*, *PAX5*	*GATA5* worse survival (*p* = 0.049)

## Supplementary Materials

The following are available online at http://www.mdpi.com/2072-6694/10/1/27/s1. Figure S1: Methylation status of the 30 TRGs in HNSCC and normal samples in TCGA database. The methylation data for 30 TRGs in HNSCC and normal samples were collected from TCGA database. * *p* < 0.05. Table S1: Q-MSP Primer List.
