# Supplementary material for: Analysis of Site-Specific Methylation of Tumor-Related Genes in Head and Neck Cancer: Potential Utility as Biomarkers for Prognosis
Source: Cancers (Basel). 2018 Jan 22;10(1):27. doi: 10.3390/cancers10010027 (PMC5789377; doi:10.3390/cancers10010027)
Supplement: Supplementary file 1 [file cancers-10-00027-s001.pdf]

Supplementary Materials: Analysis of Site-Specific Methylation of Tumour-Related Genes in Head and Neck Cancer: Potential Utility as Biomarkers for Prognosis

Kiyoshi Misawa, Daiki Mochizuki, Atsushi Imai, Masato Mima, Yuki Misawa and Hiroyuki Mineta

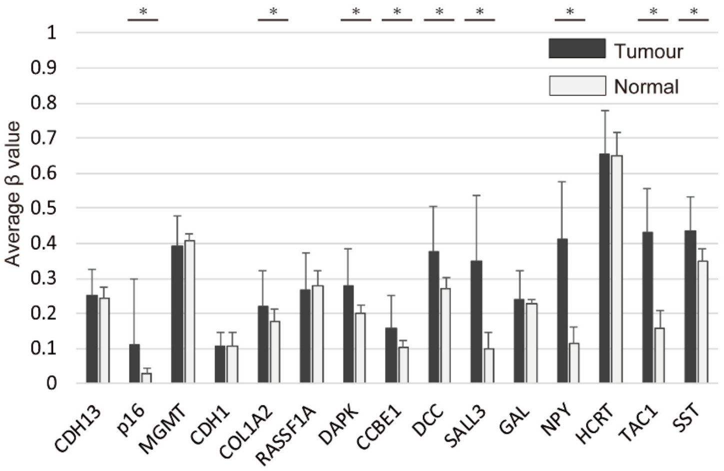

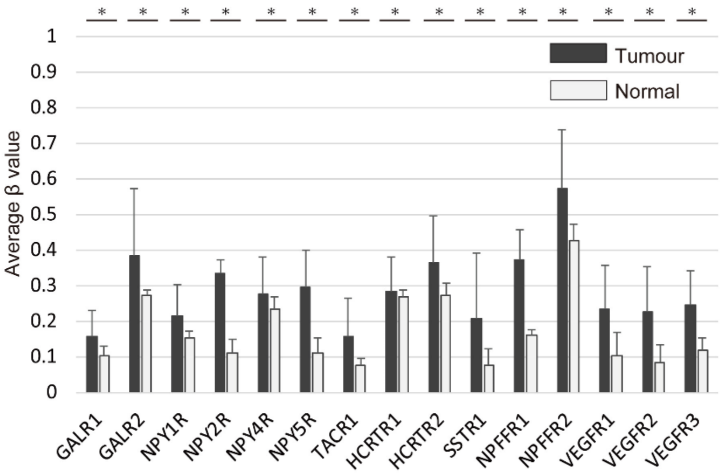

Figure S1. Methylation status of the 30 TRGs in HNSCC and normal samples in TCGA database. The methylation data for 30 TRGs in HNSCC and normal samples were collected from TCGA database. *$p < 0.05$.

Table S1. Q-MSP Primer List.

Gene	Forward primer 5′-3′	Reverse primer 5′-3′
CDH13	TTTGGGAAGTTGGTTGGTTGGC	ACTAAAAACGCCCGACGACG
p16	GTATTTTTTTCGAGTATTCGTTTACGGC	CAAATCCTCTAAAAAAAACCGCGA
MGMT	TTCGACGTTCGTAGGTTTTCGC	GCACTCTTCCGAAAACGAAACG
CDH1	GTGGGCGGGTCGTTAGTTTC	ACCACAACCAATCAACGCGA
COL1A2	ACGGTAGTAGGAGGTTTCGG	CGCAAAACCCCTAAATCACCGACG
RASSF1A	CGTTCGGTTCGCGTTTGTTAGC	TAACCCGATTAAACCCGTACTTCG

DAPK	GGATAGTCGGATCGAGTTAACGTC	CCCTCCCAAACGCCGA
CCBE1	GTCGCGGAGGAGTAGGACGCTT	CTCGAAAACGACGACACCATC
DCC	TTGTTCGCGATTTTTGGTTTC	ACCGATTACTTAAAAATACGCG
SALL3	GGGGTTCGAGCGTCGTTAGT	CCGTACTCGAAAACCCCGTC
GAL	TGACGCGATTTCGGGCGGTT	TATCCGCCGCCCGATATAAC
NPY	GTCGCGGCGAGGAAGTTTTA	ACTATACTATCGAACGAAACG
HCRT	TGATTATGGGTCGTCGCGTA	AACTATCCTCCGAACGCGAC
TAC1	GGCGGTTAATTAAATATTGAGCAGAAAGTCGC	AAATCCGAACGCGCTCTTTCG
SST	GGGGCGTTTTTTAGTTTGACGT	AACAACGATAACTCCGAACCTCG
GALR1	GGTTCGCGGTATTCGGTAGT	GGTTCGCGGTATTCGGTAGT
GALR2	CGATTGCGGGGGTTGGAGTTCGGA	CCAACAACGACCGACGACGCTA
NPY1R	TTCGGGTTCGGGTTAGCGTTG	AACGAATCTCTAACGAAACCG
NPY2R	CGAGTGAGTGCGGTGTTTAGGCG	CGAACGAACAACCGAAACAATC
NPY4R	AGGTTGGGCGGGCGTAGGCGGGA	CGAAACAAAACCGCGCCTACTT
NPY5R	AGTTACGTGTTTTCGAGACGT	CGTCCTACACCCCGACGATAA
TACR1	TTGGCGTAGTTGTCGCGTTG	CGCGAATTAACTACGCACGA
HCRTR1	CGGGTTTCGGGGTTGGAAGATA	CGGGTTTCGGGGTTGGAAGATA
HCRTR2	CGCGTAGTTTTTTTTATCGTAA	CGAACATCACGAACTCAAATCCG
SSTR1	CGGGTGCGCGAGGAGAAAGTT	TAGTTCGGGTAGTTGCGGCGAA
NPFFR1	CGTAATTAAGTGTTCGGAGATCG	CCCAATTACGAACTCCGACGA
NPFFR2	CGGCGGGTTAGTTTGGAGCG	CAACCGACGTCTATCCCCCG
VEGFR1	TGAATTTGTCGGGGTCGCGTT	TCGCTTCCCTACGACTACGAA
VEGFR2	TCGAGTTTTGGGTATTTCGTTCGGT	AACGACCCGAATCTCCACGCA
VEGFR3	GTAGCGGTCGGAGATGTAGCGGGG	ACGCTCGAACGAATAACCCGTTC

© 2018 by the authors; licensee MDPI, Basel, Switzerland. This article is an open access article distributed under the terms and conditions of the Creative Commons by Attribution (CC-BY) license (http://creativecommons.org/licenses/by/4.0/)
